# Determinants of SARS-CoV-2 Contagiousness in Household Contacts of Symptomatic Adult Index Cases

**DOI:** 10.3389/fmicb.2022.829393

**Published:** 2022-04-01

**Authors:** Mattia Trunfio, Lorenzo Richiardi, Francesca Alladio, Elena Staffilano, Bianca Longo, Francesco Venuti, Valeria Ghisetti, Elisa Burdino, Stefano Bonora, Paolo Vineis, Giovanni Di Perri, Andrea Calcagno

**Affiliations:** ^1^Infectious Diseases Unit, Amedeo di Savoia Hospital, Department of Medical Sciences, University of Turin, Turin, Italy; ^2^Cancer Epidemiology Unit, Department of Medical Sciences, University of Turin, Turin, Italy; ^3^Laboratory of Microbiology and Molecular Biology, Amedeo di Savoia Hospital, Turin, Italy; ^4^Department of Epidemiology and Biostatistics, Imperial College London, London, United Kingdom

**Keywords:** SARS-CoV-2, contagiousness, viral load, cycle threshold, transmission risk, age, household

## Abstract

**Background:**

Identifying determinants of the novel severe acute respiratory syndrome coronavirus-2 (SARS-CoV-2) transmission in settings of contagion is fundamental to inform containment strategies. We assessed SARS-CoV-2 cycle threshold value (Ct) from the first diagnostic nasal–pharyngeal swab of symptomatic index cases and which demographic or clinical characteristics among cases and contacts are associated with transmission risk within households.

**Methods:**

This is a retrospective prevalence study on secondary SARS-CoV-2 cases (SC) among the household contacts of symptomatic adult index cases randomly sampled from all the SARS-CoV-2-positive diagnostic nasopharyngeal swabs analyzed at our regional referral hospital (Amedeo di Savoia Hospital, Turin, Italy) in March, 2020. Index cases underwent a telephone survey to collect their demographic and clinical data and all their household contacts. The Ct value of RdRp gene from the first diagnostic swab of index cases was recorded and index cases were grouped according to Ct tertiles (A < first tertile, first ≤ B ≤ second tertile, C ≥ second tertile). *Post hoc* analysis was performed in SC as well as contacts that did not undergo SARS-CoV-2 testing but developed compatible signs and symptoms. Non-parametric tests and generalized linear models were run.

**Results:**

Index (*n* = 72) and contact (*n* = 164) median age was 54 (48–63) and 32 (20–56) years, respectively. A total of 60, 50, and 54 subjects were contacts of group A, B, and C index cases, respectively; 35.9% of contacts were SC. Twenty-four further subjects (14.6%) met the criteria for symptom-based likely positive SC. The secondary attack rate was 36.0% (28.6–43.4), assuming a mean incubation period of 5 days and a maximum infectious period of 20 days. SC prevalence differed between Ct groups (53.3% A, 32.0% B, 20.4% C; *p* < 0.001). No difference in SC was found according to sex, presence of signs/symptoms, and COVID-19 severity of index cases, or according to contacts’ sex and number per household. The age of both index cases [aOR 4.52 (1.2–17.0) for 60 vs. ≤45 years old] and contacts [aOR 3.66 (1.3–10.6) for 60 vs. ≤45years old] and the Ct of the index [aOR 0.17 (0.07–0.4) for Ct ≥ 31.8 vs. Ct < 24.4] independently associated with SC risk. Sensitivity analysis including symptoms-based likely positive SC supported all the previous results.

**Conclusion:**

In confined transmission settings such as households, PCR Ct values may inform on the contagiousness of infected subjects and age may modulate transmission/contagion risk.

## Graphical Abstract

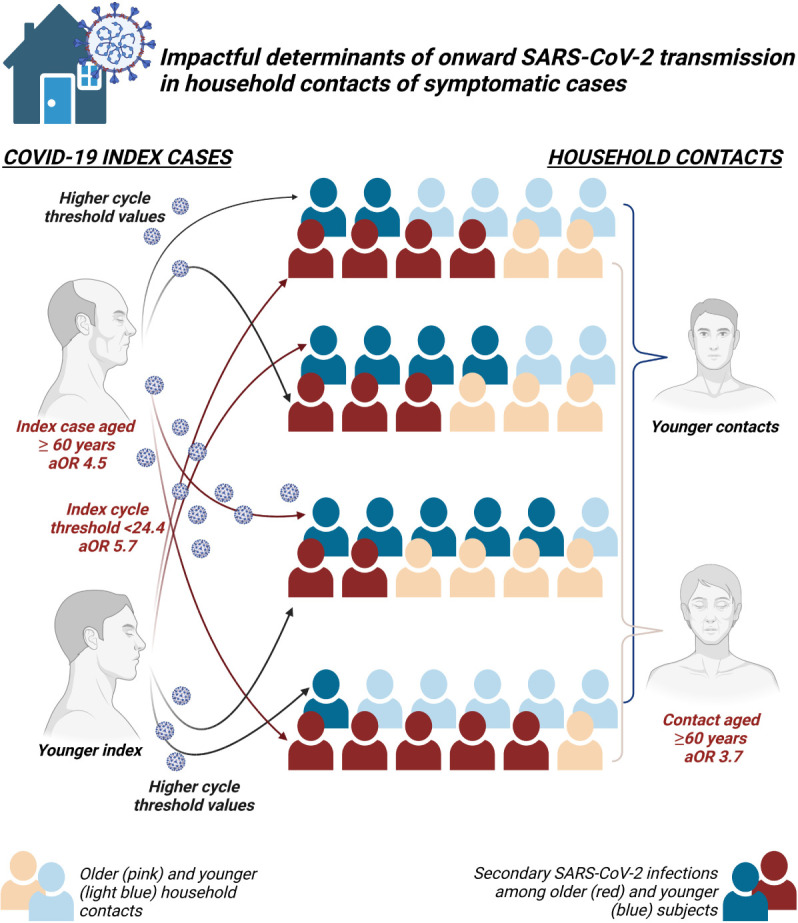

## Introduction

Since the identification of the novel severe acute respiratory syndrome coronavirus-2 (SARS-CoV-2) and the rapid rise of new cases of symptomatic disease by SARS-CoV-2 (COVID19) all over the world, it has been clear that person-to-person transmission (Lei et al., 2020; Sun et al., 2020) would have had a key role in the rapid spreading as well as in the containment of what, in March 2020, the World Health Organization declared as a pandemic.

Like most respiratory viral infections, SARS-CoV-2 spreads mainly through aerosol and droplets (Meyerowitz et al., 2020), and transmission is particularly effective in confined indoor spaces (Marks et al., 2021). Other transmission routes have been demonstrated (through fomites, *via* oral–fecal contamination, or by ocular conjunctiva), but data regarding effectiveness and eventually disease development by means of these routes are limited (Li et al., 2020; Meyerowitz et al., 2020). A similar heterogeneity has been reported to date in several viral, behavioral, social, climate, and environmental factors affecting SARS-CoV-2 transmission and subjects’ contagiousness (Carraturo et al., 2020; Koh et al., 2020; Madewell et al., 2020). Therefore, it was, and it is still clear that properly studying and defining determinants able to significantly affect SARS-CoV-2 onward transmission is challenging.

Starting from this challenge, the study of household transmission could be an easier setting to study some of such determinants while controlling for other relevant variables. Yet, so far, few studies investigated factors that may impact SARS-CoV-2 transmission risk and infectivity in this setting: conflicting lines of evidence were generated on this matter. For instance, the impact of index case age is still debated: some studies reported no impact, while others showed opposite conclusion on higher odds of transmission either among people aged less than 20 years or among subjects aged 60 years and above (Jing et al., 2020; Koh et al., 2020; Li et al., 2021; Marks et al., 2021). It is also unclear whether a higher number of household contacts can be a risk factor for increased odds of transmission (Wang et al., 2020; Li et al., 2021; Marks et al., 2021).

Furthermore, to date, scarce *in vivo* evidence points toward a higher infectivity of subjects harboring higher viral load in their upper respiratory airways (Cerami et al., 2021; Marks et al., 2021), and no virological marker has been identified for estimating the contagiousness of SARS-CoV-2-infected patients. The issue is of renewed relevance as it is not fully clear to what extent COVID-19 vaccinated subjects, because of a possible reduced viral load, have a lower probability of transmitting SARS-CoV-2 when infected compared to those who are not vaccinated (Levine-Tiefenbrun et al., 2021; Pritchard et al., 2021; Singanayagam et al., 2021; Trunfio et al., 2021d).

Herein, we sought to assess whether, among demographic and clinical parameters that may affect SARS-CoV-2 transmission within household contacts of symptomatic index cases, a virological marker represented by the inverse proxy of upper respiratory viral amount, the RT-PCR cycle threshold (Ct), associates with transmission odds and could inform about potential transmissibility from infected subjects.

## Materials and Methods

We performed a retrospective study on the risk of secondary SARS-CoV-2 cases among the household contacts of symptomatic adult patients with a SARS-CoV-2-positive diagnostic nasopharyngeal swab (NPs) analyzed by our regional reference laboratory (Amedeo di Savoia Hospital, Turin, Italy) in March, 2020.

Patients were randomly sampled from the sampling frame represented by the 1995 SARS-CoV-2-positive NPs (simple random sampling by random lottery extraction). The sampled individuals were reached in August–September 2020 for a telephone survey addressing COVID-19-related clinical and demographic characteristics of both the interviewed and all the household contacts, if any. The surveyed data were cross-checked and completed by data extrapolated using the Piedmont platform (RUPCOVID), an online regional database built for SARS-CoV-2 contact tracing, notification (NPs results and dates), and clinical data collection (demographics, signs and symptoms, date of symptoms onset). Recorded signs and symptoms were fever, asthenia, malaise, arthromyalgia, headache, olfactory and gustatory dysfunction, nausea, vomiting, diarrhea, dyspnea, runny nose, cough, and pharyngitis.

The novel severe acute respiratory syndrome coronavirus-2 was isolated from 300 μl of nasopharyngeal and oropharyngeal swabs (NPs) collected in Universal Transport Medium for viruses^®^ (UTM, Copan group, Brescia, Italy). Viral RNA was extracted using the NucliSENS easyMag instruments (bioMérieux, Italia Spa, Bagno a Ripoli, Italy), with a final elution volume of 50 μl. Five microliters of RNA was processed with the 2019 Novel Coronavirus Real-Time Multiplex PCR (RT-PCR) kit (Liferiver Bio-Tech, San Diego, CA, United States), a multiplex TaqMan real-time PCR that targets the following three SARS-CoV-2-specific genes: ORF1ab (RdRp), nucleocapsid (N), and envelope (E). For the purpose of the study, only RdRp Ct values were considered to have one uniform proxy of viral load. All reactions were performed in 7500 Fast thermal cycler (Applied Biosystems, San Diego, CA, United States). RT-PCR results were analyzed with the ABI PRISM manager software. SARS-CoV-2-positive (mixture of plasmids containing partial RdRp, N, and E fragments) and -negative controls were used for each run. The procedure included an internal control added into the specimen before the RNA extraction phase, to evaluate RNA extraction efficiency and the presence of inhibitors. SARS-CoV-2 RT-PCR results were considered positive if at least two target genes were amplified with a Ct of ≤40, inconclusive if only one target was amplified with a Ct of ≤40, and negative if no gene targets were amplified or amplified with a Ct of >40 after background subtraction. Ct was defined as the number of cycles of amplification required for the fluorescence of SARS-CoV-2 PCR to be detected above the background signal and can be used as a relative inverse proportional measure of viral amount in the specimen. RT-PCR sensitivity was tested against dilution series of virus spiked into negative samples, and the 95% lower detection limit was determined by probit analysis. For NPs, the 95% limit of detection was 324, 213, and 365 copies/ml for the RdRp, N, and E genes, respectively, as per manufacturer instructions. Specificity of the RT-PCR procedure has been verified for non-specific reactions to other human endemic coronaviruses (HCoV) 229E, NL63, OC43, and HKU1 as well as MERS-CoV using supernatants from cell culture and none of the virus preparations showed reactivity for any of the three genes of the assay, as per manufacturer evaluation.

Data from interviewed subjects meeting the definition criteria of index case and living with at least another subject were used for the study. Index case was defined as any adult subject with a proved SARS-CoV-2 infection (NPs with RdRp Ct value < 40) developing COVID-19-related signs and/or symptoms at least 5 days before any other subjects living in the same house (according to the median incubation time) (Wassie et al., 2020). Sampled subjects meeting the definition of index case but had NPs collected at emergency departments or were already hospitalized were excluded to include only index cases with COVID-19 onset and relevant attendance at home. Households with asymptomatic secondary cases of index cases for whom an extra-household source of infection was not disclosed (e.g., index cases reporting testing due to contacts with known positive subjects outside the household) were excluded; this was settled to reduce potential classification bias in cases with no datable COVID-19 onset.

The tertiles of the distribution of Ct values from the first diagnostic swab were used as cutoffs for grouping index cases as follows: Ct < first tertile, group A; first tertile ≤ Ct < second tertile, group B; Ct ≥ second tertile, group C.

Household contacts were defined as swab-confirmed secondary cases when the following criteria were met: living in the same household of the index case, of any age, with a SARS-CoV-2-positive NPs and, if symptomatic, with the onset of COVID-19 at least 5 days after the onset in the linked index (as per index case definition) and, if asymptomatic, with the first positive NPs collected at least 5 days after the onset of the linked index case. Household contacts developing COVID-19-related signs and symptoms or testing positive with or without disease more than 25 days (20 + 5) from the COVID-19 onset of the index case were not considered as secondary cases (according to available data on wild-type SARS-CoV-2 length of contagiousness potential in cohort including severe disease or immunocompromised subjects + median incubation period) (Singanayagam et al., 2020; Wassie et al., 2020; Johansson et al., 2021; Owusu et al., 2021; van Kampen et al., 2021).

A symptom-based PCR strategy could miss up to 62% of SARS-CoV-2 infections, as recently reported in a large prospective cohort (Ng et al., 2021). *Post hoc* analysis was performed including also symptoms-based likely positive secondary cases (defined as contacts living in the same household of the index case, of any age, who did not undergo SARS-CoV-2 testing or tested negative but eventually developed SARS-CoV-2-related signs or symptoms at least 5 days after the onset in the index case), to assess whether missing testing for SARS-CoV-2 in some of the household contacts may have introduced bias.

Informed consent was asked and obtained during the phone survey; those not consenting were not included and their data were not collected. Pseudo-anonymized data were used. The study was conducted according to the guidelines of the Declaration of Helsinki and approved by the Inter-departments Ethics Committee A.O.U. Città della Salute e della Scienza, A.O. Ordine Mauriziano di Torino, and A.S.L. Città di Torino (Torino, Italy, protocol number 0065839-00304/2020, July 9, 2020).

For descriptive purposes, we reported the variable distributions of the household contacts and the index subjects separately in each of the three Ct groups of index cases. Categorical variables were presented as absolute numbers and proportions, continuous variables were presented as median and interquartile range, and their univariable association with the three Ct groups was tested using non-parametric tests (Mann–Whitney, Chi-square test for trend, and Kruskal–Wallis). To further investigate the relationship between the Ct value of index cases and prevalence of secondary cases among household contacts, we ran a generalized linear model (log regression for binary outcomes), with robust variance and clustering at the household level, which included variables with univariate *p*-value < 0.10 adjusted for age of both index and contacts. The analyses were clustered by household variable to adjust for household-related variables not collected during the survey. The secondary attack rate (SAR) was estimated as the proportion of susceptible household contacts that were identified as secondary SARS-CoV-2 cases (for both swab-confirmed and swab-confirmed plus symptoms-based likely positive definitions) during the 25 days after COVID-19 onset in the index case. Data analysis was performed using Stata SE 16 (StataCorp LLC, TX, United States).

## Results

Two-hundred and thirty subjects were sampled, and 200 consented to the survey; among these, 72 subjects met the criteria for index case and were included in the analyses together with their 164 household contacts.

The mean SAR (95% confidence interval) among the 72 household clusters was 36.0% (28.6–43.4%) when considering swab-confirmed secondary cases and 55.5% (47.9–63.1%) when including symptoms-based likely positive secondary cases.

Among the index cases, 47 subjects (65.3%) were male and 71 (98.6%) were of European ancestry, with a median age, Ct at diagnosis, number of household contacts, and time from disease onset to diagnostic swab of 54 years (48–63), 28.81 (22.24–33.64), 2 (1–3), and 7 days (3–10), respectively. Forty-eight index cases (66.7%) required hospital admission for oxygen support due to moderate or severe COVID-19; 24.41 and 31.79 were the first and second tertile of the Ct distribution, resulting in 24 index cases (33.3%) per group as per study methods. Comparisons between index groups in terms of demographic, clinical, and household contact characteristics are shown in Table 1. The index groups differed in terms of severity of COVID-19 (requirement of oxygen support) and temporal gap between COVID-19 onset and diagnostic swab collection only (Table 1).

**TABLE 1:** Comparison of demographic, clinical, and household characteristics between contact cases (upper table) and between index cases (lower table).

Household contacts	*A (n = 60)*	*B (n = 50)*	*C (n = 54)*	*p*
Age, median years (IQR)	31 (16–55)	39 (21–55)	32 (22–61)	0.906
Male, *n* (%)	29 (48.3%)	14 (28.0%)	22 (40.7%)	0.094
Caucasian, *n* (%)	57 (95.0%)	48 (96.0%)	52 (96.3%)	0.938
**Secondary swab-confirmed COVID-19 cases, *n* (%)**				
Among all contacts	32 (53.3%)	16 (32.0%)	11 (20.4%)	***0.001***
Among tested contacts only	32/38 (84.2%)	16/29 (55.2%)	11/26 (42.3%)	***0.002***
Secondary swab-confirmed plus symptom-based likely COVID-19 cases, *n* (%)	44 (73.3%)	25 (50.0%)	22 (40.7%)	***0.001***

**Index cases**	***A (n = 24)***	***B (n = 24)***	***C (n = 24)***	***p***

Ct value, median (IQR)	19.83 (18.71–22.23)	28.81 (27.22–30.78)	34.71 (33.79–36.08)	**–**
Age, median years (IQR)	57 (42–65)	53 (48–59)	56 (50–61)	0.178
Male, *n* (%)	13 (54.2%)	19 (79.2%)	15 (62.5%)	0.184
Caucasian, *n* (%)	24 (100%)	23 (95.8%)	24 (100%)	0.368
Contacts per household, median (IQR)	3 (1–3)	2 (1–3)	2 (1–3)	0.481
Tested contacts per household, median (IQR)	1 (1–2)	1 (1–1)	1 (0–2)	0.632
Tested contacts/overall contacts per household, median (IQR)	87.5 (33.3–100)	100 (33.3–100)	66.7 (33.3–100)	0.411
Time from COVID-19 onset to swab collection, median days (IQR)	3 (2–6)	9 (6–10)	7 (3–10)	***0.001***
Oxygen requirement due to COVID-19, *n* (%)	12 (50.0%)	19 (79.2%)	17 (70.8%)	***0.053***
With any transmission symptoms*, *n* (%)	11 (45.8%)	13 (54.2%)	11 (45.8%)	0.803
With respiratory transmission symptoms only*, *n* (%)	10 (41.7%)	13 (54.2%)	9 (37.5%)	0.486

*Transmission symptoms were considered: vomiting, diarrhea, runny nose, and cough.

*Respiratory transmission symptoms were considered: runny nose and cough; Ct, Cycle threshold; COVID-19, symptomatic SARS-CoV-2 infection; IQR, interquartile range. p-values statistically significant (<0.05).*

Among household contacts, 65 (39.6%) were male and 148 (90.2%) were of European ancestry, with a median age of 32 years (20–56). There were 60 (36.6%), 50 (30.5%), and 54 (32.9%) contacts of the group A, B, and C, respectively. Ninety-three (56.7%) underwent SARS-CoV-2 nasal–pharyngeal swab (60 were positive), of whom 59 (35.9%) met the definition of secondary case [median difference in COVID-19 onset between secondary and index cases of 5 days (5–6)].

Among contacts that did not undergo testing (*n* = 71, 43.2%), 24 (14.6% of the overall contacts) had signs and symptoms suggestive of COVID-19. All of them met the criteria for symptom-based likely positive secondary case [median difference in time of COVID-19 onset between secondary and index cases of 6 days (5–7)]. Eight further patients who underwent swab collection and had negative results but subsequently developed suggestive signs or symptoms were also considered as symptom-based likely positive COVID-19 cases.

As for potential confounders, contacts’ age, ethnicity, and sex, the number of household contacts, and the proportion of contacts undergoing swab collection did not differ between the index groups (Table 1).

**FIGURE 1:**
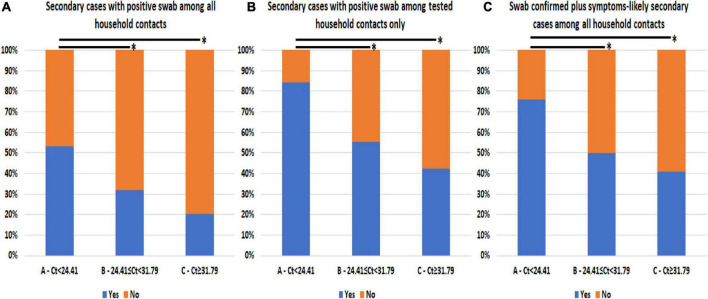
Prevalence of secondary SARS-CoV-2 infections among household contacts according to cycle threshold (Ct) values from the diagnostic nasal–pharyngeal swab of linked index cases. **(A)** Swab-confirmed secondary cases among all the household contacts stratified according to index first Ct value. **(B)** Swab-confirmed secondary cases among only the household contacts that underwent testing stratified according to index first Ct value. **(C)** Swab-confirmed secondary cases plus symptoms-based likely positive secondary cases among all the household contacts stratified according to index first Ct value. *Comparison with *p*-value < 0.05 is highlighted.

The prevalence of secondary SARS-CoV-2 infections among household contacts differed according to the Ct value detected in the diagnostic swab of the linked index case, as shown in Table 1 and Figure 1A. Group A had a higher prevalence of secondary cases (53.3%) compared to groups B (32.0%, *p* = 0.025) and C (20.4%, *p* < 0.0005). Considering only household contacts who underwent swab testing, group A (84.2%) had more secondary cases than groups B (55.2%, *p* = 0.010) and C (42.3%, *p* = 0.001; Figure 1B). Similar results were observed when symptom-based likely positive secondary cases were included in the analysis: group A (73.3%) observed more secondary cases compared to groups B (50.0%, *p* = 0.012) and C (40.7%, *p* < 0.0005; Table 1 and Figure 1C).

**FIGURE 2:**
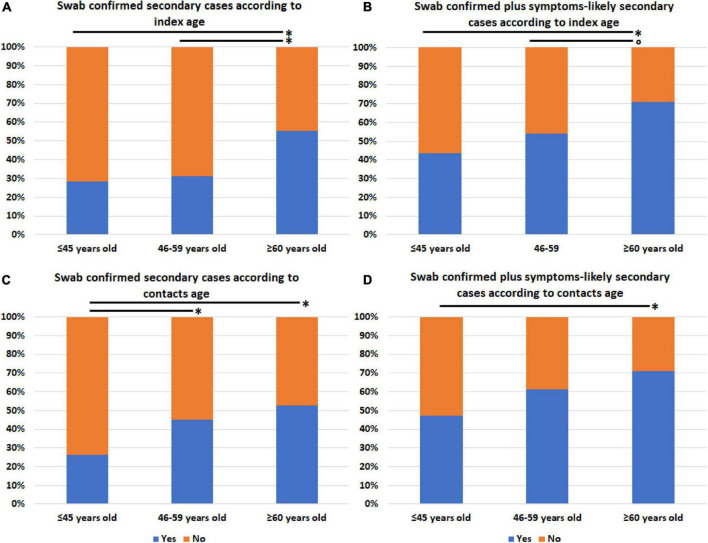
Prevalence of secondary SARS-CoV-2 infections among household contacts according to age of either linked index cases or contacts. **(A)** Swab-confirmed secondary cases among all the household contacts according to index case age. **(B)** Swab-confirmed plus symptoms-based likely positive secondary cases among all the household contacts according to index case age. **(C)** Swab-confirmed secondary cases among all the household contacts according to contacts’ age. **(D)** Swab-confirmed plus symptoms-based likely positive secondary cases among all the household contacts according to contacts’ age. *p-value ≤ 0.05; trend for statistical significance, *p*-value of 0.076.

We also assessed whether the prevalence of secondary cases (both swab positives only and swab positives plus symptom-based likely positives) differed according to age, sex, presence of signs, or symptoms potentially related to increased emission of virions by droplet/aerosol particles or fomite contamination, and COVID-19 severity of linked index cases or to age, sex, and number of household contacts (1, 2, 3, or more than three members) regardless of Ct stratification. No difference was observed between the groups identified according to all the considered variables (data not shown), except for both index and contacts’ age stratified into three groups (≤45, 46–59, and ≥60 years old). In fact, the prevalence of swab positives only and swab positives plus symptom-based likely positive secondary cases significantly increased with increasing age of both index cases (Figures 2A,B) and household contacts (Figures 2C,D).

In multivariable models, index and contacts’ age as well as Ct values of the index were the only independent variable associated with the risk of swab-confirmed secondary cases and swab-confirmed plus symptoms-based likely positive secondary cases within households (Table 2). Specifically, older age of both contacts and index cases increased the risk of SARS-CoV-2 transmission by about three times more in both models for subjects aged 60 years and above compared to 45 years old subjects or younger (Table 2), while higher Ct values decreased the odds of transmission by 83 and 87% when group C was compared to group A in the two models, respectively.

**TABLE 2:** Multivariate models for factors associated with differential risk of secondary SARS-CoV-2 cases among household contacts.

Variable	aOR (95%CI)	*P*
**GLM for eventually testing positive at SARS-CoV-2 swab among household contacts**		
**Index age**		
≤45 years old	Ref.	–
46–59 years old	1.96 (0.61–6.29)	0.255
≥60 years old	4.52 (1.20–17.03)	***0.026***
Index case requiring oxygen (ref. not requiring)	0.47 (0.17–1.29)	0.145
**Index PCR Cycle threshold value (Ct)**		
A, Ct < 24.41	Ref.	–
B, 24.41 ≤ Ct < 31.79	0.38 (0.13–1.11)	0.077
C, Ct ≥ 31.79	0.17 (0.068–0.44)	**<*0.0005***
Time from COVID-19 onset to swab collection in index cases	0.99 (0.89–1.09)	0.824
**Contact age**		
≤45 years old	Ref.	–
46–59 years old	2.84 (1.22–6.64)	***0.016***
≥60 years old	3.66 (1.27–10.55)	***0.016***
Contact sex (ref. male)	1.57 (0.66–3.75)	0.312

**GLM for eventually testing positive at SARS-CoV-2 swab or developing suggestive symptoms/signs among household contacts**		

**Index age**		
≤45 years old	Ref.	–
46–59 years old	3.39 (1.21–9.50)	***0.020***
≥60 years old	4.14 (1.17–14.61)	***0.027***
Index case requiring oxygen (ref. not requiring)	1.13 (0.39–3.26)	0.814
**Index PCR Ct value**		
A, Ct < 24.41	Ref.	–
B, 24.41 ≤ Ct < 31.79	0.18 (0.049–0.63)	***0.008***
C, Ct ≥ 31.79	0.13 (0.043–0.40)	**<*0.0005***
Time from COVID-19 onset to swab collection in index cases	1.02 (0.92–1.14)	0.673
**Contact age**		
≤45 years old	Ref.	–
46–59 years old	1.95 (0.81–4.65)	0.134
≥60 years old	2.96 (1.11–7.93)	***0.030***
Contact sex (ref. male)	0.72 (0.31–1.63)	0.427

*p-values statistically significant (<0.05).*

## Discussion

Among household clusters during the beginning of the pandemic in a north-western city of Italy, we observed a higher odds of SARS-CoV-2 onward transmission as age of both symptomatic index cases and contacts increased as well as lower odds of transmission as the Ct value from the first diagnostic nasal–pharyngeal swab of index cases increased. The hierarchy of the impact on transmission potential was dominated by Ct values, followed by the age of index cases and of household contacts. Number of contacts, presence of signs and symptoms suggestive of potential higher spread of infectious virions (such as runny nose, cough, and diarrhea), the sex of contacts and index, as well as the overall COVID-19 severity of the index cases were not found to independently affect transmission risk in the same setting.

Our study increases the amount of emerging evidence in support of a significant dose–contagiousness relationship in SARS-CoV-2 transmission dynamics and it positions among those studies in favor of a relevant role of age of both infected and receptive subjects in transmission odds.

Whether the dose–infectivity relationship follows an exponential, linear, or logarithmic scale is not clear, and we could not address this question with our study design. Our data provide additional support to the association between PCR Ct values and SARS-CoV-2 transmission risk that few other groups have observed both in households and in settings with similar prolonged and close contacts such as school and workplace (Kawasuji et al., 2020; Al Bayat et al., 2021; Cerami et al., 2021; Marks et al., 2021). SARS-CoV-2 RT-PCR is the gold standard diagnostic method for SARS-CoV-2 infection as per international guidelines, but Ct assessment still shows several pre-analytical (intrinsic variability on the operator and the tolerance of the patients during swab collection) and analytical issues (there is variability in the starting amount of template that inversely correlates with the Ct threshold for each sample, and the use of raw Ct values can understate the dispersion of the measurements). In this regard, we have tried to limit (pre-)analytical variability using a sampling frame of swabs all analyzed in the same laboratory, with the same assay and during a 1-month period. Nevertheless, our data aim at supporting evidence on the relationship between the viral amount of the source and the risk of onward transmission among contacts, which is still debated, but we agree with the Infectious Diseases Society of America, the Association of Molecular Pathology, and the American Association for Clinical Chemistry that recommend against using Ct values to guide patient management due to the several limitations associated with reporting Ct values.

Of note, viral load of the infecting source also seems to predict COVID-19 severity and long-term sequelae (Shah et al., 2021; Trunfio et al., 2021c), and a debate on whether they may associate with severity among secondary cases is ongoing. Nevertheless, to date, both *in vivo* and *in vitro* data point toward no evidence for such an association (Brosseau et al., 2021; Cerami et al., 2021; Trunfio et al., 2021a,b). It would be advisable to improve an international standardization of assessing and reporting Ct values or SARS-CoV-2-RNA viral load, when available, to improve the reliability of these tools in routine applications, to provide further data characterizing the type of mathematical functions regulating the relationship between viral amount, contagiousness, and other SARS-CoV-2 infection-related features, and perhaps to identify reliable cutoffs able to distinguish different risk categories. The second tertile of the Ct distribution of our population (31.79) does not diverge so far from what *in vitro* models detected as a potential cutoff for viral infectivity from human samples to cell cultures, approximately 30–35 (Jefferson et al., 2020; La Scola et al., 2020; Singanayagam et al., 2020; Al Bayat et al., 2021), and from a recent study reporting a 1.5 increased risk among close contacts of index with Ct < 30 compared to those of index with higher Ct (Al Bayat et al., 2021). Viral load and cell culture infectivity cannot be translated directly to *in vivo* contagiousness, but the similarity between the *in vitro* studies and our real-life data adjusted for several variables that cannot be included in culture models strengthens the evidence of a significant drop in transmission potential (of more than 80%) when index sources show early Ct values approaching 30–35 or greater values.

The relationship between viral load of the source and the odds of infection in contacts also has further implications. First, this finding may add a piece to the ongoing debate on whether breakthrough SARS-CoV-2 infections among vaccinated subjects are less contagious (Singanayagam et al., 2021). As evidence on faster declining or reduced peak viral load among breakthrough infections is mounting (Jacobson et al., 2021; Levine-Tiefenbrun et al., 2021; Pritchard et al., 2021; Singanayagam et al., 2021), further data demonstrating a dose–infectiousness relationship such as ours could endorse the theory that vaccinated subjects may be less contagious also due to a reduction in their median-in-time viral load. Secondly, proper call to maintaining protective measures even within household should be implemented as face masking can significantly reduce transmission by modulating viral shedding (Goyal et al., 2021), but fatigue and adherence to such preventive behaviors may more easily fade in this setting as the pandemic continues (Carlucci et al., 2020). Besides, considering the early transmission occurrence, further studies are required to assess the timing of implementation (as well as SARS-CoV-2 positivity notification) and the most effective preventive practices in similar settings.

The association between the age of index cases and infectious potential as well as between the age of contacts and contagion odds is controversial. Some authors reported no association with index’s age (Madewell et al., 2021), while others observed SARS-CoV-2 transmission odds either increasing or decreasing with age of index (Goldstein et al., 2021; Li et al., 2021; Lee et al., 2022) or contacts only (Jing et al., 2020; Goldstein et al., 2021; Li et al., 2021; Marks et al., 2021). This inconsistency may depend on whether children, asymptomatic infections, and different transmission settings were included in the studies.

The explanation for what we and others have observed (Li et al., 2021; Marks et al., 2021) may depend on both biological and social factors. Age has been described to tightly correlate with SARS-CoV-2 viral amount, ACE2-receptor expression/density, and comorbidities, and these last with each other (Baker et al., 2021; Euser et al., 2021; Fagyas et al., 2021; Trunfio et al., 2021c); therefore, index and contacts’ age may represent merely a proxy of viral amount shedding and receptors’ availability, respectively. Nevertheless, we found that age of both contacts and index may be associated with onward transmission independently from the source’s viral load, and this could be related to the potential different type of contacts and inter-personal dynamics within households that elderly people have compared to younger subjects. Indeed, the elderly may require more frequent and longer assistance with proximity physical contacts when chronically or acutely sick. In a household setting, this can be difficult to delegate or avoid despite recommendations on self-isolation among either caregivers or old subjects in need of assistance.

Higher SARs of symptomatic index cases compared to asymptomatic cases have been reported (Koh et al., 2020; Madewell et al., 2021). While expectoration has been occasionally associated with an increased risk of transmission (Luo et al., 2020), in agreement with our findings and previous negative findings (Madewell et al., 2021; Marks et al., 2021), if SARS-CoV-2 infection becomes clinically manifest, it seems that further stratifications according to the presence/absence of specific signs and symptoms or disease severity do not associate with differential odds of transmission in confined settings. These negative findings may suggest a transmission model within the household that could witness a quick attainment of the maximum infectivity potential, where the biologically plausible contribution of increased dispersion of virions may be nuanced by more impactful determinants and that symptomatic cases are more likely to transmit before symptoms onset (Li et al., 2021).

Our study presents several limitations, such as the limited sample size and the retrospective design. As for the latter, while prospective studies can better address outcomes such as secondary infection rate, there are very few prospective studies with a sufficiently long follow-up to detect all the actual secondary events. Despite the fact that our data collection at 6 months can be exposed to recall bias, it allowed for a more reliable recollection of all the secondary infections. Furthermore, we have corrected our model for the time between COVID-19 onset and swab collection to adjust for temporal variations in viral amount.

We did not collect data on comorbidities, domestic relations, or features of the home environment, and our population was mainly represented by white/Caucasian subjects; all these variables may affect transmission dynamics. We partially limited this data gap by clustering the analyses at the household level and including age (proxy of comorbidity) in the model.

Our study population belongs to March 2020 and considered only symptomatic index; therefore, any conclusion on transmission determinants may not apply to asymptomatic infections, SARS-CoV-2 variants of concerns, or current households where naturally or vaccine-induced immunized people and proper adherence to clear transmission mitigation strategies should modify what we have described in a naïve population exposed to wild-type virus free of behaving and circulating as naturally as possible. While preliminary findings align with ours (Marc et al., 2021; Lee et al., 2022), further data on the predictive role of initial viral load in the onward transmission of SARS-CoV-2 variants of concerns are warranted. Lastly, 23.8% of the overall population of contacts could have included asymptomatic infections that were not detected by our assessment strategy and criteria for secondary cases.

We have also observed a relatively high SAR (36.0%). While it may have been underestimated due to the only partial contact testing, an overestimation could have been introduced too, as we cannot rule out that some of the classified secondary infections were incubating the infection for a longer period than the attributed index. However, the detected SAR is in line with other reports during the same period and setting in countries heavily hit during the first wave as Italy, when no previous seroprotection, vaccination, and adherence to certain protective measures were in place (Boscolo-Rizzo et al., 2020; Koh et al., 2020; Cerami et al., 2021). Furthermore, this value could be explained by the longer temporal criteria we adopted to define secondary infections (within 25 days) compared to prospective studies with shorter follow-up.

## Conclusion

Identifying determinants that play a role in the risk of transmitting and/or acquiring SARS-CoV-2 is relevant to inform and execute public health strategies; reducing the transmission chain using tailored strategies may be needed in order to save human and economic resources while improving effectiveness. For this purpose, the role of viral load quantification from early diagnostic NPs in differentiating infectiousness potential deserves further studies beyond the assessment of a proxy variable such as Ct values, as well as the reasons underlying higher transmissivity and sensitivity to SARS-CoV-2 infection among older infected subjects and contacts, respectively.

## Data Availability Statement

The raw data supporting the conclusions of this article will be made available by the authors, without undue reservation.

## Ethics Statement

The studies involving human participants were reviewed and approved by the Regional Inter-company Department for Infectious Diseases and Emergency (DIRMEI, Torino, Italy). Written informed consent to participate in this study was provided by the participants’ legal guardian/next of kin.

## Author Contributions

MT: conceptualization, data curation, formal analysis, investigation, methodology, project administration, software, validation, visualization, and roles and writing—original draft. LR: conceptualization, data curation, formal analysis, investigation, methodology, supervision, validation, and writing—review and editing. FA, BL, and FV: data curation, investigation, methodology, writing—review and editing. ES: conceptualization, roles and writing—original draft, and writing—review and editing. VG: data curation, formal analysis, investigation, methodology, supervision, validation, and writing—review and editing. EB: data curation, formal analysis, investigation, methodology, validation, and writing—review and editing. SB: conceptualization, project administration, supervision, and writing—review and editing. PV: supervision and writing—review and editing. GD: conceptualization, methodology, project administration, supervision, and writing—review and editing. AC: conceptualization, data curation, investigation, methodology, project administration, supervision, validation, and writing—review and editing. All authors contributed to the article and approved the submitted version.

## Conflict of Interest

The authors declare that the research was conducted in the absence of any commercial or financial relationships that could be construed as a potential conflict of interest.

## Publisher’s Note

All claims expressed in this article are solely those of the authors and do not necessarily represent those of their affiliated organizations, or those of the publisher, the editors and the reviewers. Any product that may be evaluated in this article, or claim that may be made by its manufacturer, is not guaranteed or endorsed by the publisher.
